# Development of African Forum for primary health care

**DOI:** 10.4102/phcfm.v13i1.2973

**Published:** 2021-04-13

**Authors:** Shabir Moosa

**Affiliations:** 1 Department of Family Medicine and Primary Care, Faculty of Health Sciences, University of the Witwatersrand, Johannesburg, South Africa; 2 WONCA Africa, Johannesburg, South Africa

World Organisation of Family Doctors (WONCA) Africa has been able to use these editorials to share a basic understanding of WONCA Africa as an organization,^1^ some plans and achievements since 2018^2^ and its growing relationship with World Health Organization African Region (WHO AFRO).^3^ An important project of WONCA Africa is the development of the African Forum for Primary Healthcare (AfroPHC). It speaks strongly to the Kampala Commitment.^4^

As a result of the growing relationship between WONCA Africa and WHO AFRO, Dr Prosper Tumusiime joined the WONCA Africa Conference of June 2019 in Kampala, Uganda. We had a workshop with Dr Tumusiime on building the collaboration between WONCA Africa and WHO AFRO. In exploring the opportunities, Prof. Jan de Maeseneer suggested developing AfroPHC, along the lines of the European Forum for Primary Care (EFPC). The EFPC was set up in 2005 as a forum of primary care (PC) providers to promote strong PC in Europe.

This idea was explored further in the second Interprofessional Education and Collaborative Conference for Africa of the Africa Interprofessional Education Network (AfrIPEN) held in Nairobi, Kenya in August 2019. Africa Interprofessional Education Network is a partnership between various institutions and individuals seeking to establish interprofessional education and collaborative practice (IPE) as an integral part in training the health workforce and in the effective functioning of systems for health in sub-Saharan Africa. The meeting was opportune as AfrIPEN had a multitude of various health professionals in its midst and had established a relationship with WHO AFRO, with Dr Tumusiime being at the conference as well. The idea of AfroPHC was nurtured with various leaders engaged, especially Dr Tumusiime of WHO AFRO, Dr Champion Nyoni of AfrIPEN and Ms Bongi Sibanda, leading the African Advanced Practice Nursing initiative. There was great enthusiasm, some basic ideas and other African primary healthcare groups and leaders were identified.

A short document sharing the vision for AfroPHC and a planned face-to-face gathering in June 2020 was developed amongst identified leadership of African frontline primary healthcare worker organisations in the context of the high-level Universal Health Coverage (UHC) Declaration in September 2019. The vision essentially was to advocate for appropriate PHC and UHC in Africa by bringing together the leadership of all healthcare workers at the coalface of African Primary Care (PC) and ensure that we had a voice in policy on PC/PHC in Africa. The plan was to develop AfroPHC as the voice of the PC/PHC team and its supporters, sharing and supporting each other in advocating for PHC. The strategy was to engage each other using a Google Group and plan a face-to-face workshop in June 2020. There was instant enthusiasm with a wide range of relevant organisations joining the call, including WONCA Africa (for family doctors), African Network of Associate Clinicians (for clinical officers/associates), Anglophone African Advanced Practice Network (AAAPN Coalition) (for family nurse practitioners), International Council of Nurses (ICN) (for nurses generally), Towards Unity For Health (for public health practitioners), AFREHealth (for health educators/researchers in Africa), AfrIPEN (for allied health professions and interprofessional practice), Primafamed (for family medicine educators), African Medical and Research Foundation (AMREF) (for CHWs/community stakeholders), West African Institute of Public Healthand its Academy (for public health) and WHO AFRO. They formed the AfroPHC Core Team.

Whilst the planned face-to-face AfroPHC workshop in June 2020 did not materialise because of coronavirus disease 2019 (COVID-19), there were a number of achievements over 2020. These PHC organisations (and a number of global supporters e.g. PHCPI) met monthly online as the AfroPHC Core Team. There were a series of webinars from February to May 2020 allowing the different organisations to share more about themselves and their constituencies in Africa. AfroPHC was also part of a series of webinars on COVID-19 responses. A collaboration with AMREF resulted in the offering of the online Leadership, Management and Governance of Health Systems Strengthening Course to AfroPHC constituents, garnering more than 200 participants. More recently there is a collaboration with the World Continuing Education Council to provide continuing professional development courses to constituencies. There are over 600 members of the AfroPHC Google Group, serving as a communication vehicle for African PHC stakeholders.

The AfroPHCcore team has gelled African teamwork amongst PHC team leaders but visible PHC teamwork across Africa started with the first AfroPHC workshop during 9–11 September 2020. Dr Tumusiime, of WHO AFRO, opened the workshop. Various PHC leaders then shared their thoughts over the 3 hour sessions on getting to know the PHC Team in Africa, what the community expects from PHC in Africa and how the PHC team should function in Africa. It was billed by participants as brilliant and one of its kind, especially for its online participation and interactivity. The workshop emerged with an AfroPHC Statement that laid out key principles for the organisation. It stressed the nature of PHC as people-centred, with PHC human resources, capacity development, teamwork, inclusive PHC leadership and advocacy as key.

The resulting AfroPHC Statement was released at the AfroPHC for UHC Workshop on 10th December 2020. Mr. Jim Campbell (Director Workforce at WHO) and Dr Suraya Dalil (Director PHC at WHO) were part of the panel in this workshop who reviewed the statement and reflected on workforce issues for PHC in Africa. This workshop also considered the development of AfroPHC as a formal organisation. A report on the UHC Workshop was released. AfroPHC will be launched as a formal organisation on 20 April 2021.

What has become plain is that PHC teamwork is taking quick shape at a high level in Africa. The various deliberations have shown that we seem to share more ideas and values than we had expected. It is very possible that we will be able to produce a document on PHC Teamwork in Africa that will be tabled with WHO AFRO and that will help define the development of PHC and UHC in Africa.

WONCA Africa provides considerable leadership and material support to the development of AfroPHC. It is heart-warming to know that this leadership by WONCA Africa is respected by all the various leaders of the PHC team in Africa. WONCA Africa and Primafamed have even been drawn into development of the Advanced Practice Nurse Training Framework in Africa. The strong cohesion of AfroPHC is respected by many stakeholders, with AfroPHC (and WONCA Africa) being drawn into a number of pan-African and global forums as an organisation that stakeholders are eager to listen to.

AfroPHC demonstrates an attribute that family physicians need to nurture as part of their daily PHC teamwork: the best leaders are those who build the team around to function as leaders themselves.
